# Supplementary material for: Orbital perspective on high-harmonic generation from solids
Source: Nat Commun. 2023 Dec 18;14:8421. doi: 10.1038/s41467-023-44041-0 (PMC10728088; doi:10.1038/s41467-023-44041-0)
Supplement: Supplementary file 1 — Supplementary Information [file 41467_2023_44041_MOESM1_ESM.pdf]

Supplementary information. Orbital perspective on high-harmonic generation from solids.

Á. Jiménez-Galán[1,2,3], Chandler Bossaer[1,4], Guilmot Ernotte[1], Andrew M. Parks[4], Rui E.F. Silva[3], David M. Villeneuve[1], André Staudte[1], Thomas Brabec[4], Adina Luican-Mayer[4], and G. Vampa[1]

[1] *Joint Attosecond Science Laboratory, National Research Council of Canada and University of Ottawa, Ottawa, ON K1A 0R6, Canada.*
[2] *Max-Born-Institute, Max-Born Strasse 2A, D-12489, Berlin, Germany.*
[3] *Instituto de Ciencia de Materiales de Madrid (ICMM), Consejo Superior de Investigaciones Cientficas (CSIC), Sor Juana Ins de la Cruz 3, 28049 Madrid, Spain.*
[4] *Department of Physics, University of Ottawa, Ottawa, ON K1N 6N5, Canada.*

Supplementary note 1. Band structure.

Fig. S1 shows the band structure along different directions.

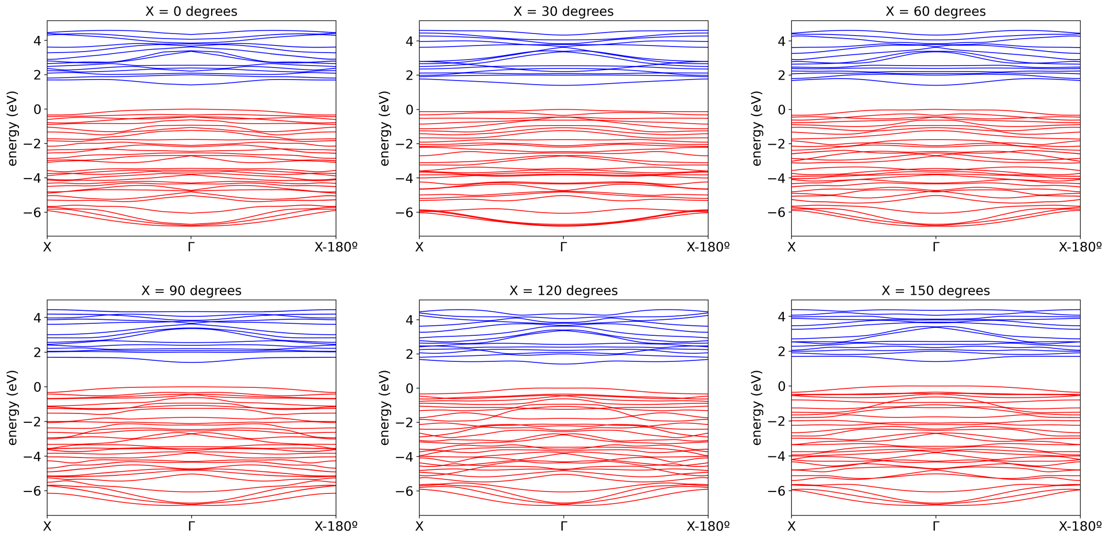

Supplementary Figure 1: Band structure of monolayer ReS_2 along different angles with respect to the a-axis of the crystal (see Fig.1 in main text).

Supplementary note 2. Fourier amplitudes and phases.

Here, we provide an orbital-based analysis of the angular dependence of H9, analogous to that of H11 in the main text. We first compare the coherent and incoherent sum of the orbital currents, shown in Fig. S2. We observe that both are very similar for the case of low intensity (blue curve). In this case, the signal is dominated by emission along the parallel component to the driving field (Fig. S3). For this component, the biggest Fourier amplitude comes from the atomic pair Re_1-Re_3 at $\theta = 100°$ (Fig. S5, blue circles), which explains the maximum observed at this angle in the Fig.3a of the main text (blue curve) and Fig. S2 (faint dashed blue curve). In this case, phase interference plays minimal role since the phase spread is not too large across different angles (Fig. S5).

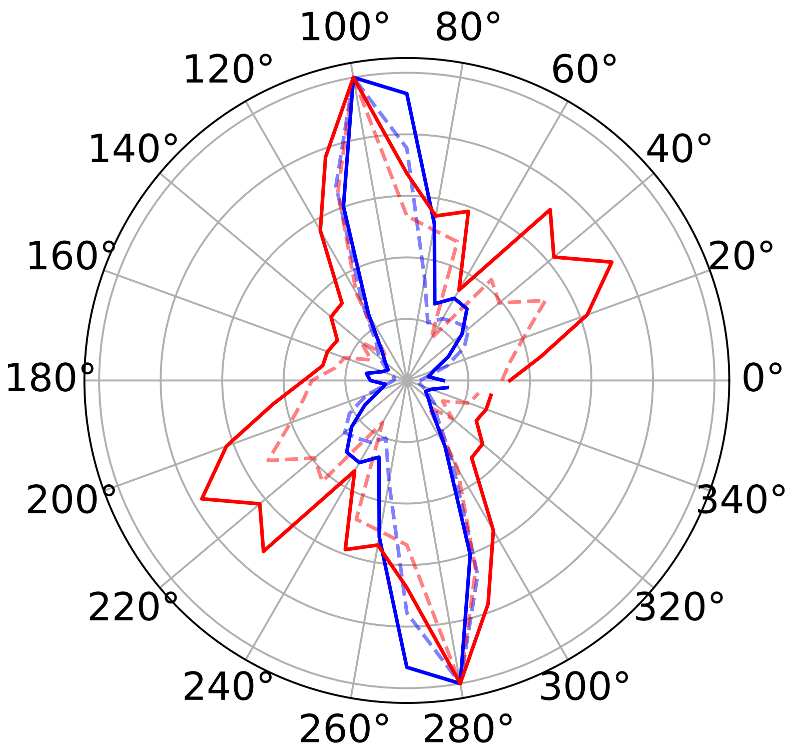

Supplementary Figure 2: Calculation neglecting the Fourier phase (solid lines) φ_n of the orbital current for H9 for 0.1 TW/cm^2 (blue) and 0.6 TW/cm^2 (red). For comparison, the full calculation curves of main text Fig.3a are shown in with dashed, faint lines.

For the highest intensity (red curve), both the coherent and incoherent sum show a peak at 100°. While that may seem to suggest that orbital phase interference also does not play a role here, it is not the case. Indeed, Fig. S4 shows that the total signal for H9 at 100° is dominated by the emission along the parallel component to the driving field. However, the Fourier amplitudes for parallel emission are small for 100° (see Fig. S7), but they do have the least spread of the phases. Why does the signal maximize at 100° in the incoherent case then? The answer is in the perpendicular emission (see Fig. S8), which does have maximum amplitudes at this angle (Fig. S8). Hence, the total signal, which is dominated by the parallel component, has a maximum at 100° because of the orbital phase interference, even if the maximum for the coherent and incoherent cases coincide. On top of this, such phase interference is also responsible for the pronounced suppression of the maximum seen in the incoherent case at 40°.

Figs. S3,S4 show the harmonic angular dependence resolved in both parallel and perpendicular components with respect to the driving field for H3-H13. Fig. S5-S8 show the Fourier amplitudes and phases for H3-H13, along both the parallel and perpendicular component with respect to the driving field, and for low and high

intensity.

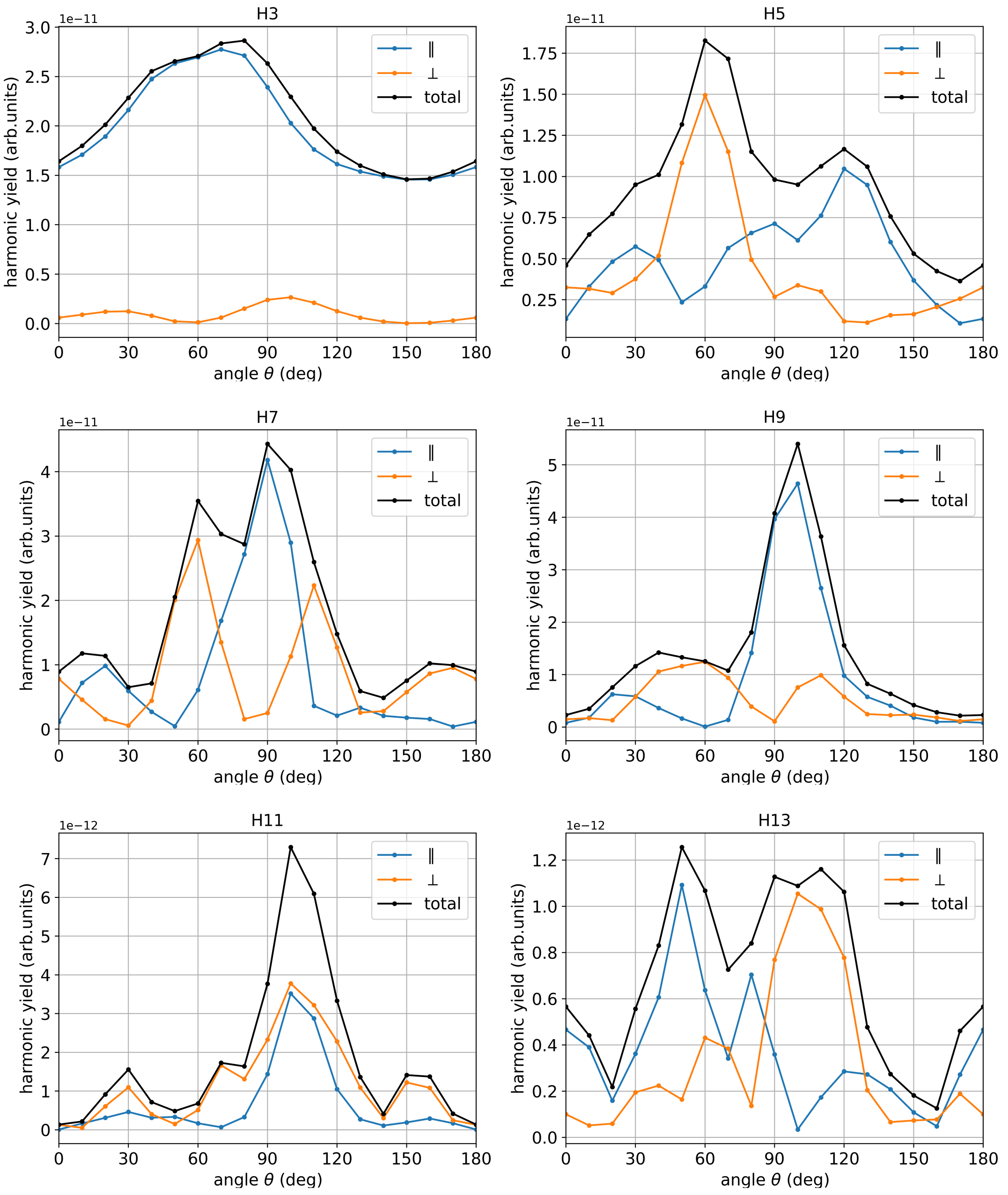

Supplementary Figure 3: Angular dependence of harmonics 3-13 resolved in parallel (blue) and perpendicular (orange) components with respect to the driving field. The total signal (sum of both components) is shown in black. The intensity of the driving field is $I = 0.1$ TW/cm^2.

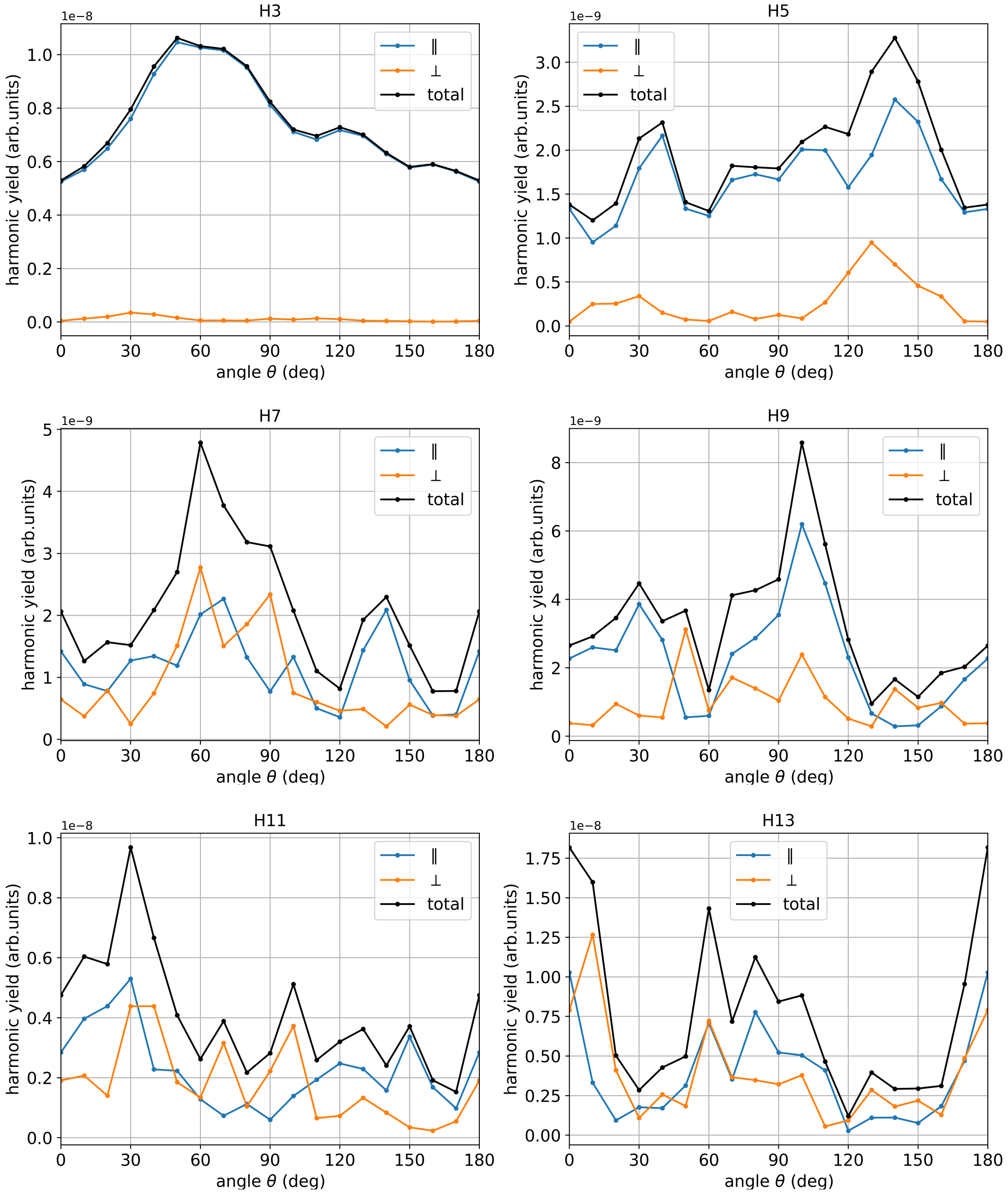

Supplementary Figure 4: Angular dependence of harmonics 3-13 resolved in parallel (blue) and perpendicular (orange) components with respect to the driving field. The total signal (sum of both components) is shown in black. The intensity of the driving field is $I = 0.6$ TW/cm^2.

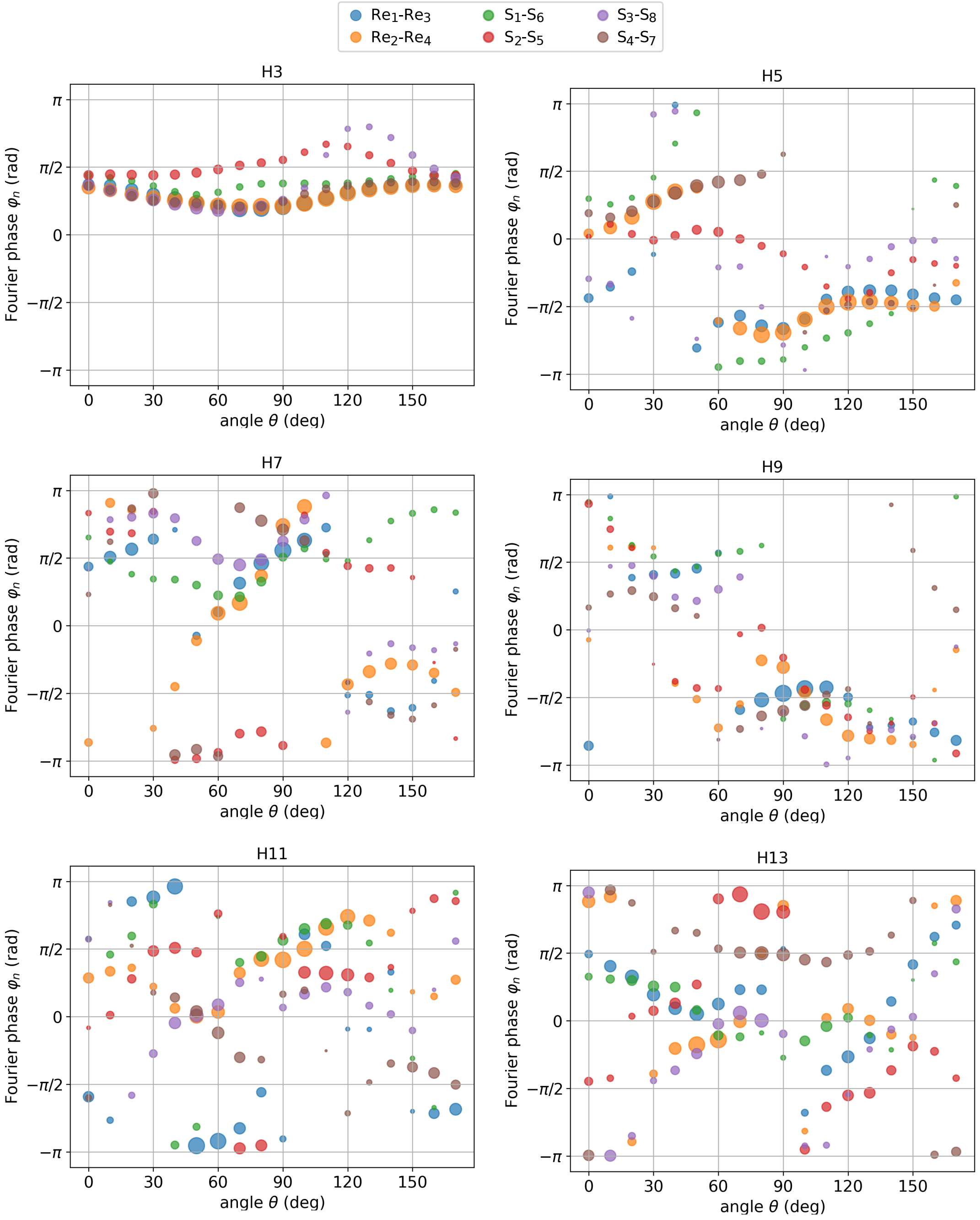

Supplementary Figure 5: Fourier amplitudes and phases for harmonics 3-13 along the component parallel to the driving field. The intensity is $I = 0.1$ TW/cm^2.

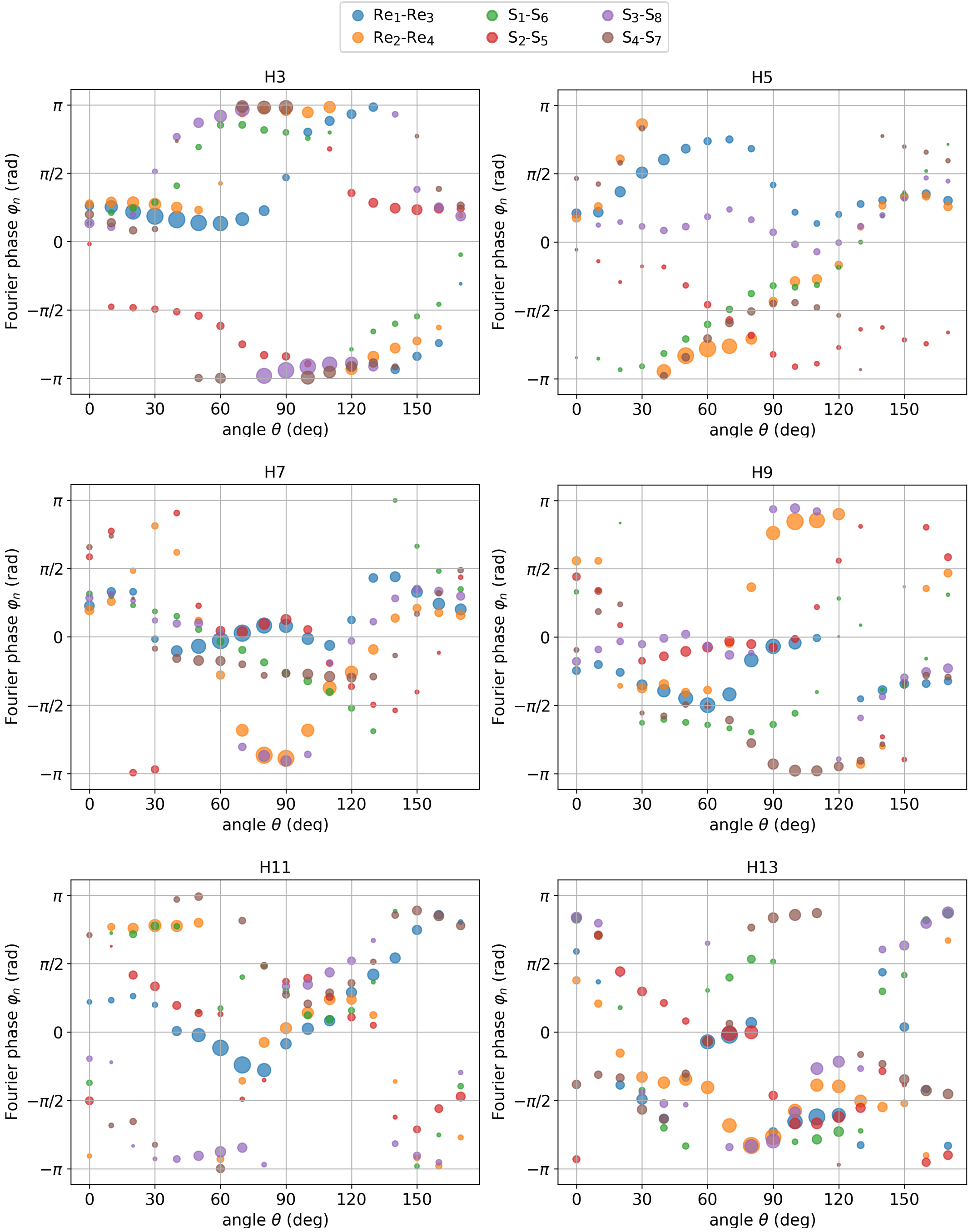

Supplementary Figure 6: Fourier amplitudes and phases for harmonics 3-13 along the component perpendicular to the driving field. The intensity is I = 0.1 TW/cm^2.

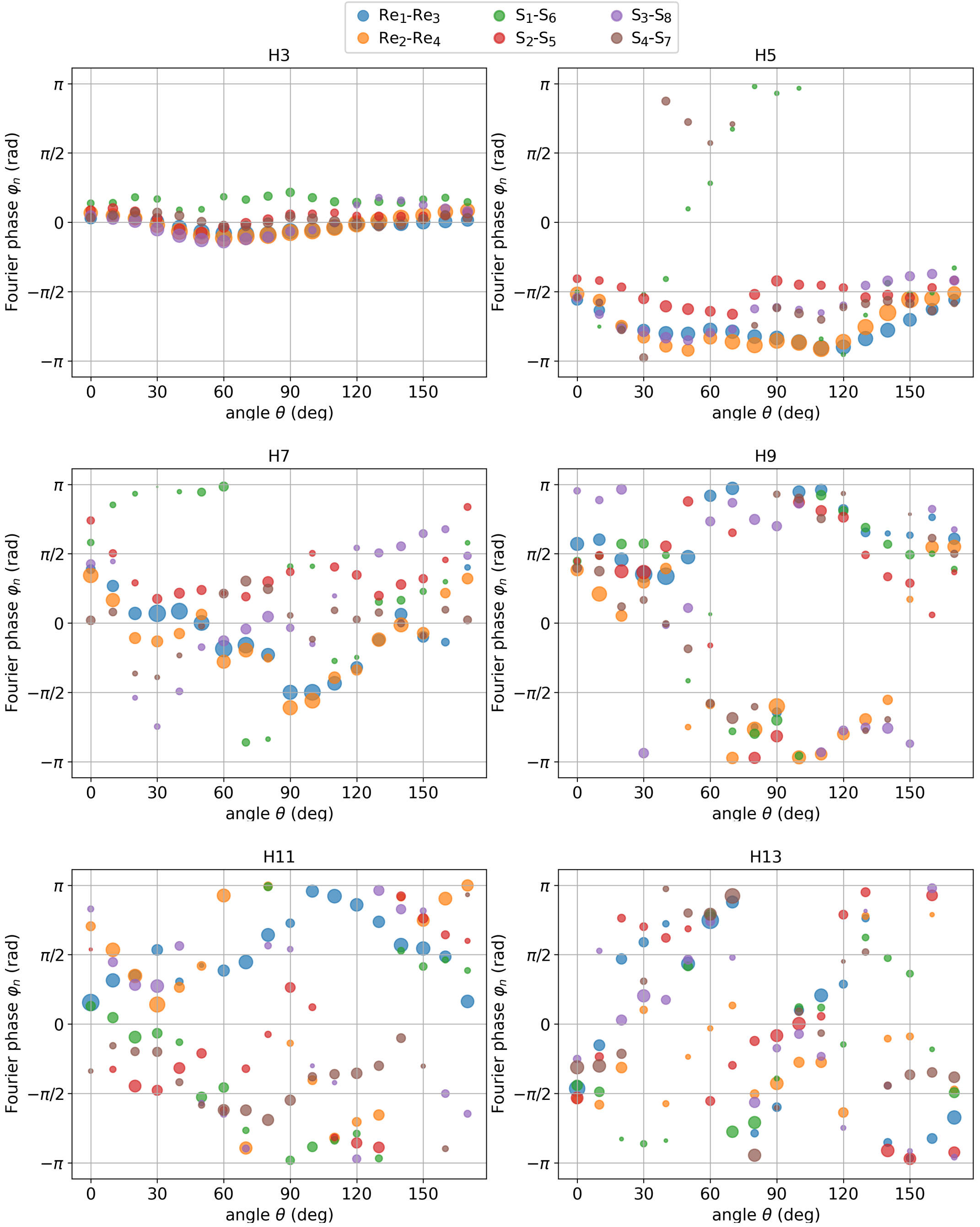

Supplementary Figure 7: Fourier amplitudes and phases for harmonics 3-13 along the component parallel to the driving field. The intensity is $I = 0.6$ TW/cm^2.

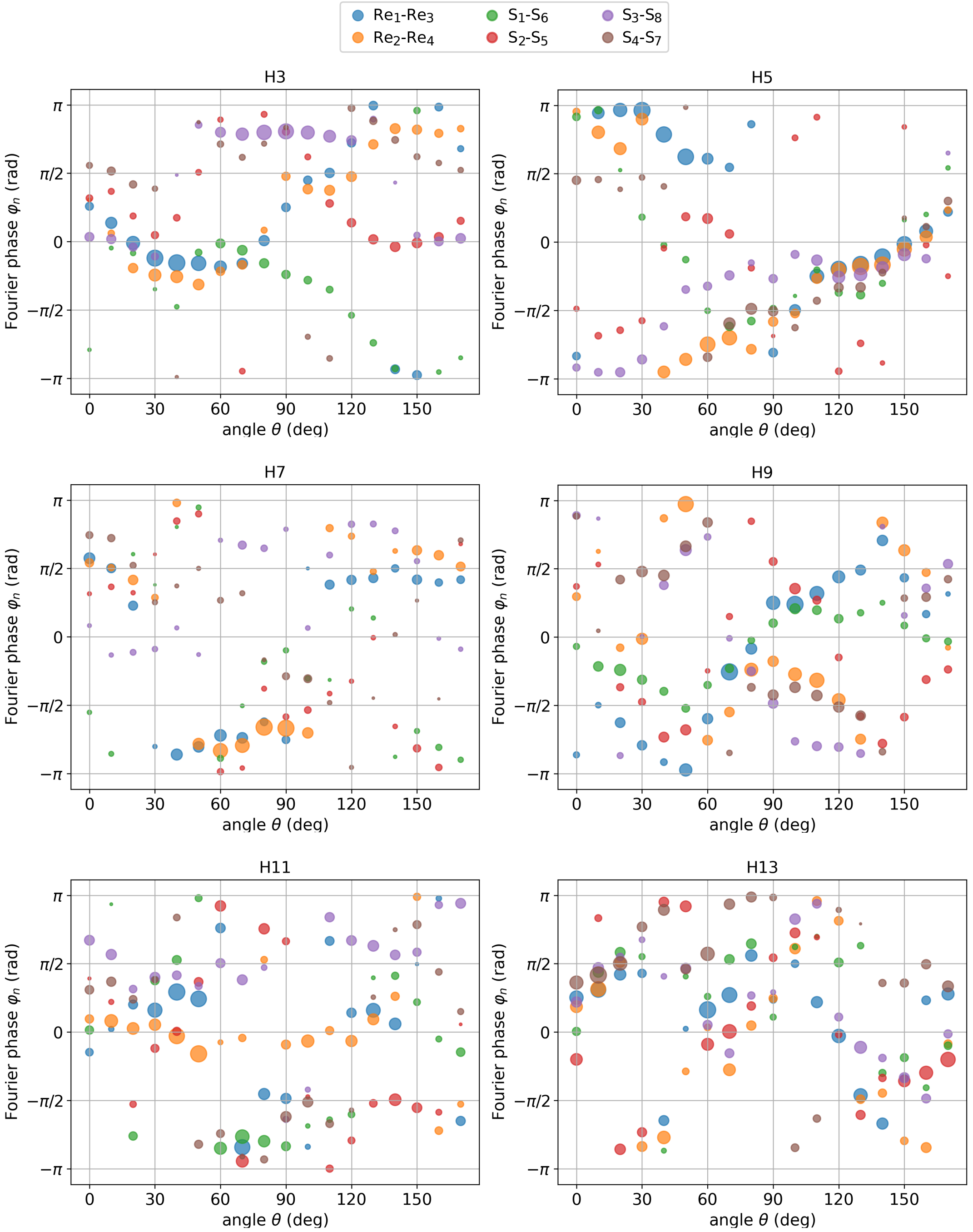

Supplementary Figure 8: Fourier amplitudes and phases for harmonics 3-13 along the component perpendicular to the driving field. The intensity is $I = 0.6$ TW/cm^2.

Figs. S9-S12 show the mean and standard deviation of the phase at each angle, weighted by the amplitudes of the orbitals.

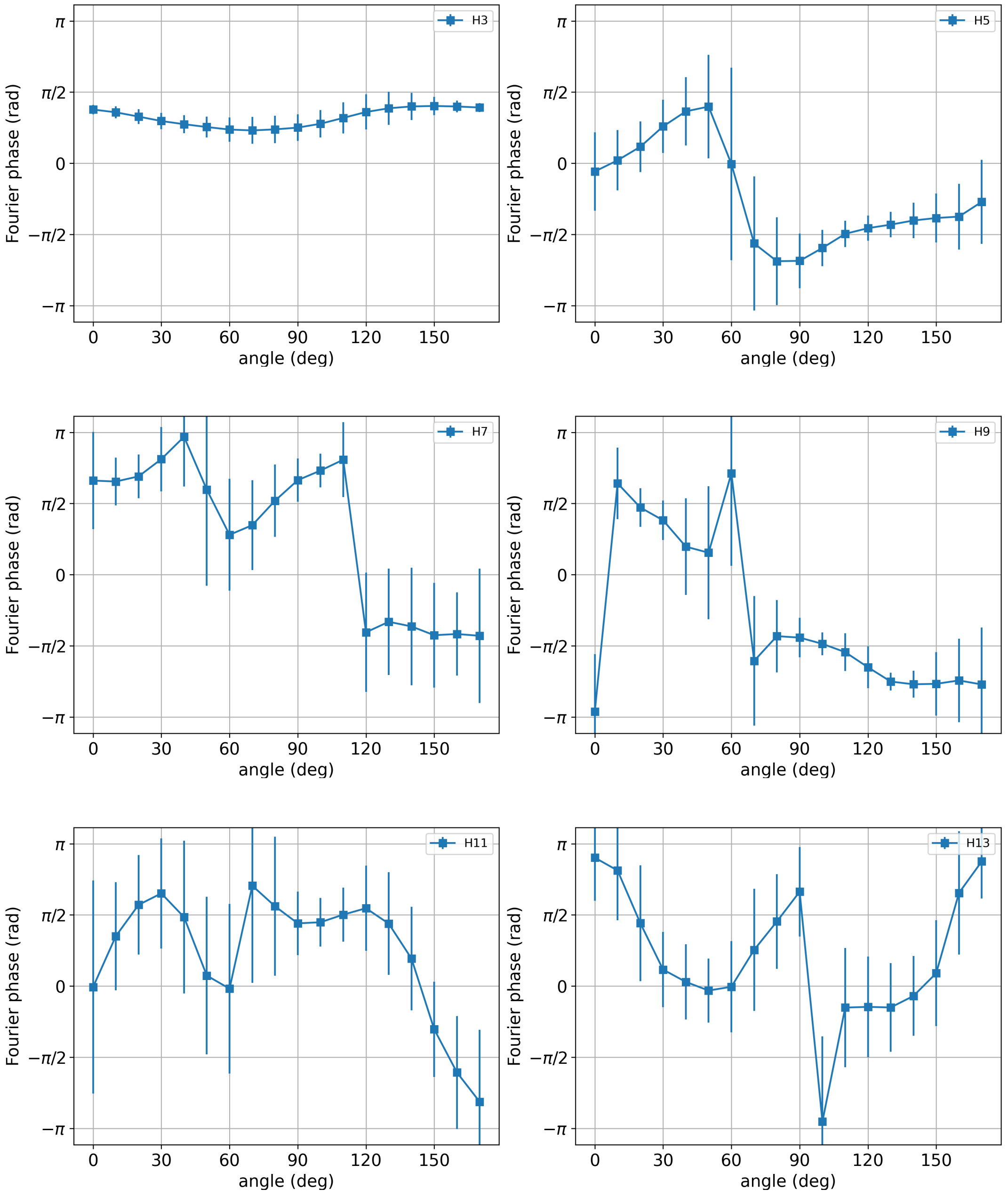

Supplementary Figure 9: Weighted mean and standard deviation of the Fourier phase for harmonics 3-13, weighted by the amplitudes of the orbitals. The intensity is $I = 0.1$ TW/cm^2 and the component is parallel to the polarization of the driving field.

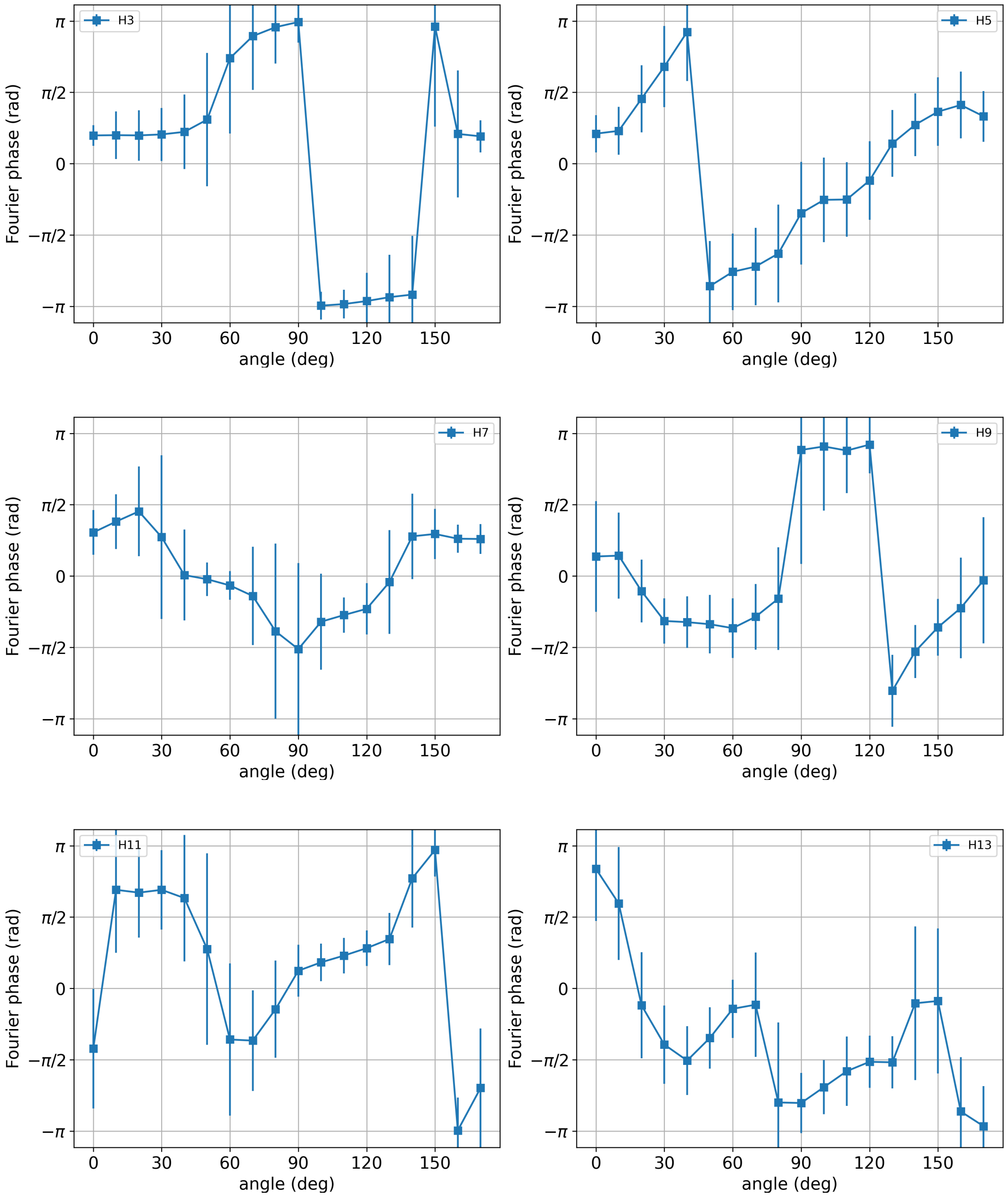

Supplementary Figure 10: Weighted mean and standard deviation of the Fourier phase for harmonics 3-13, weighted by the amplitudes of the orbitals. The intensity is $I = 0.1$ TW/cm^2 and the component is perpendicular to the polarization of the driving field.

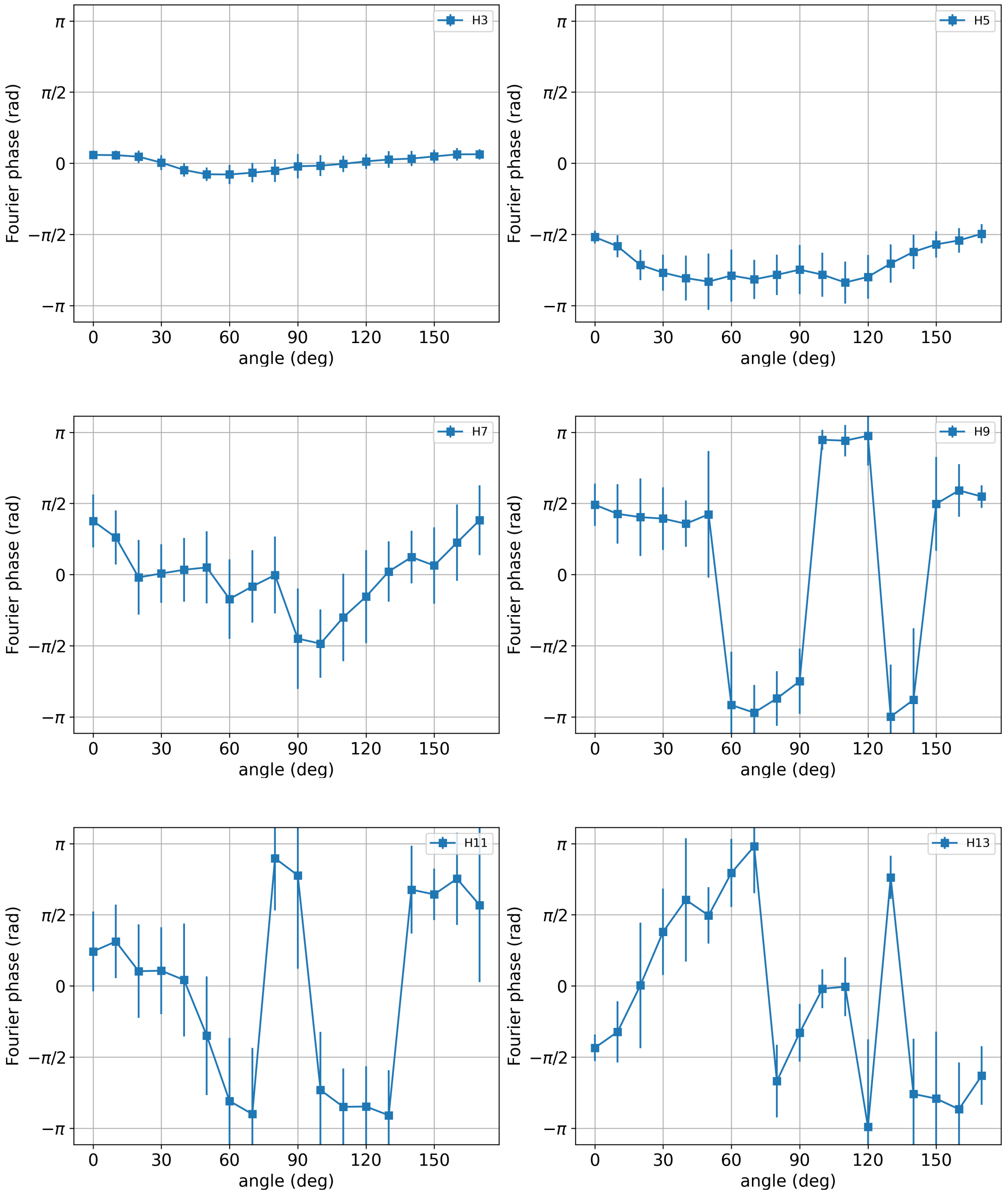

Supplementary Figure 11: Weighted mean and standard deviation of the Fourier phase for harmonics 3-13, weighted by the amplitudes of the orbitals. The intensity is $I = 0.6$ TW/cm^2 and the component is parallel to the polarization of the driving field.

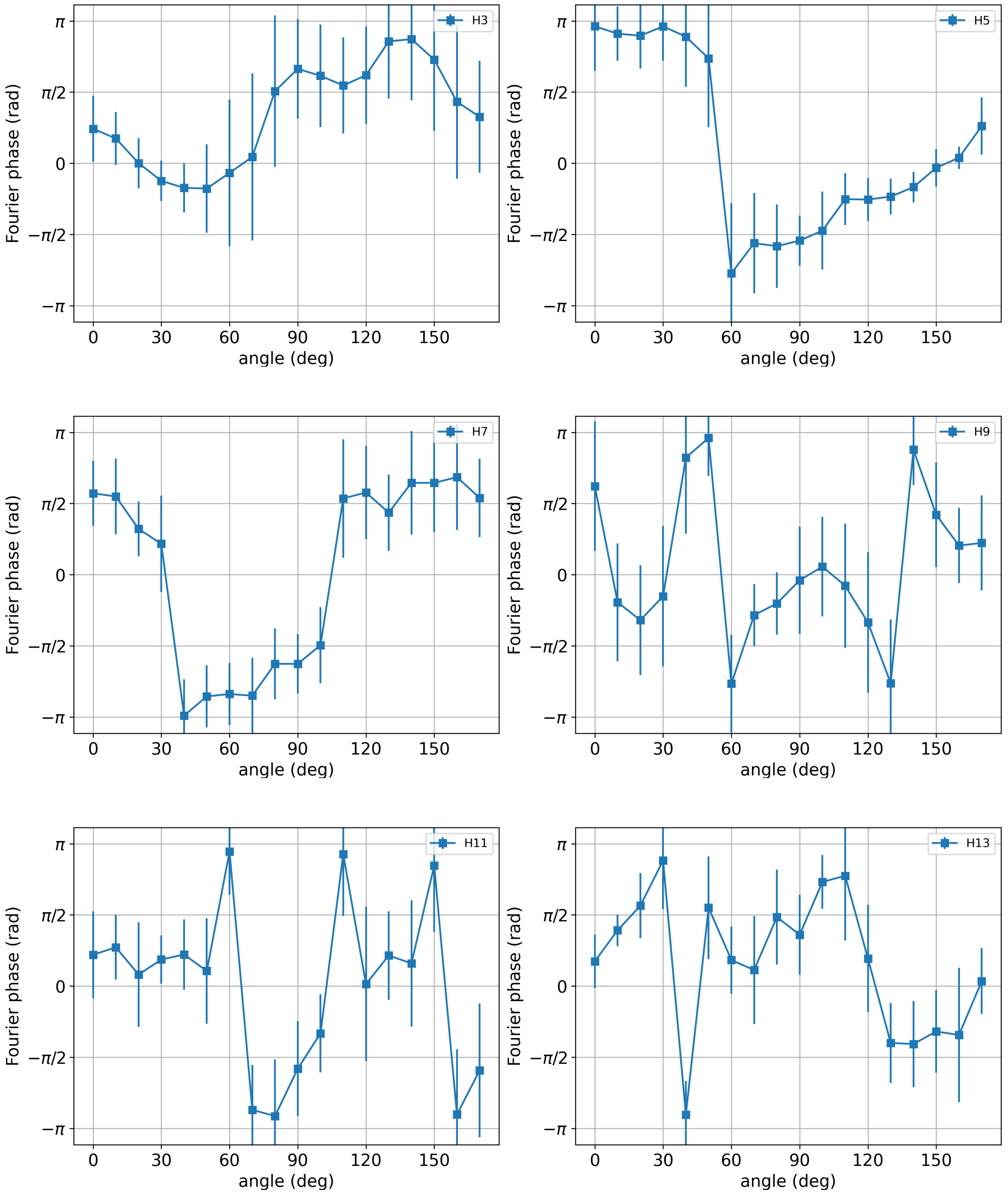

Supplementary Figure 12: Weighted mean and standard deviation of the Fourier phase for harmonics 3-13, weighted by the amplitudes of the orbitals. The intensity is $I = 0.6$ TW/cm^2 and the component is perpendicular to the polarization of the driving field.

Supplementary note 3. Influence of dephasing and detuning of central wavelength on angle-resolved HHG spectrum.

To show the impact of dephasing, we have made calculations for three different dephasing times: T_2 = 2 fs, T_2 = 5 fs and T_2 = 10 fs. Results are shown in Fig. 13, showing that the exact position of the peaks changes, but a strong anisotropy is observable for all choices of T_2.

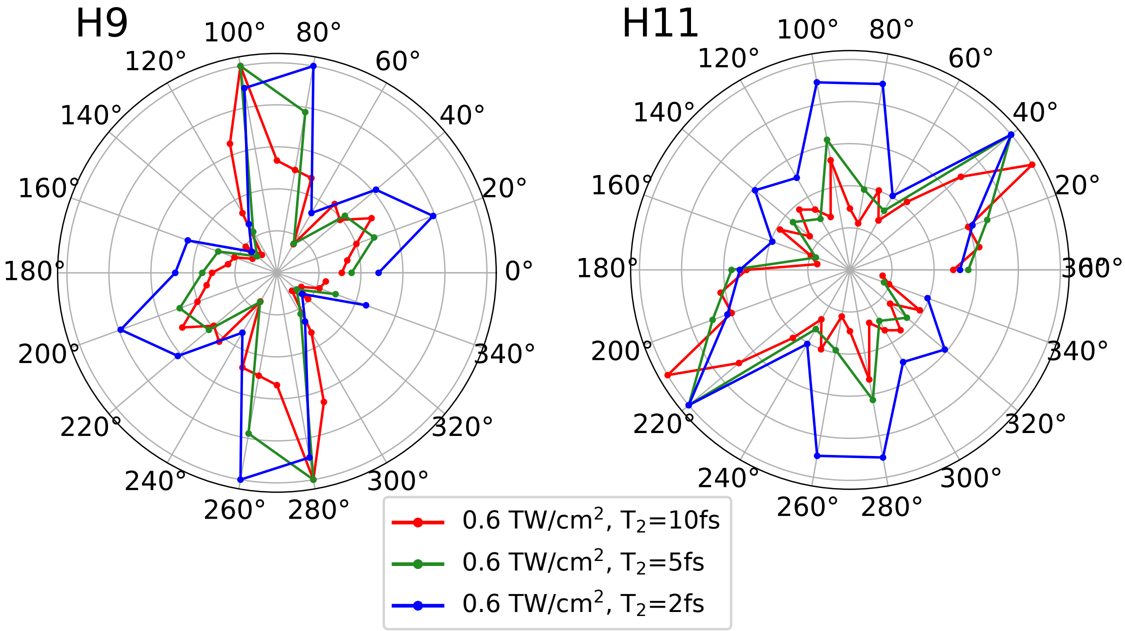

Supplementary Figure 13: Dependence of dephasing on angle-resolved HHG in ReS_2. Simulated angle-resolved, energy-integrated spectrum for harmonics 9 (left) and 11 (right) for different dephasing parameters: T_2 = 10 fs (red curve), 5 fs (green curve) and 2 fs (blue curve). The intensity is fixed at I = 0.6 TW/cm^2.

In Fig. 14 we show how the angle-dependent HHG yield also changes for a detuning of the laser central wavelength (3.5 μm) of $\simeq$ 6%. As the results show, even slight changes in the energy scales of the system can affect the exact positions of the peaks in the angle-resolved HHG, but a strong anisotropy is observable for all cases.

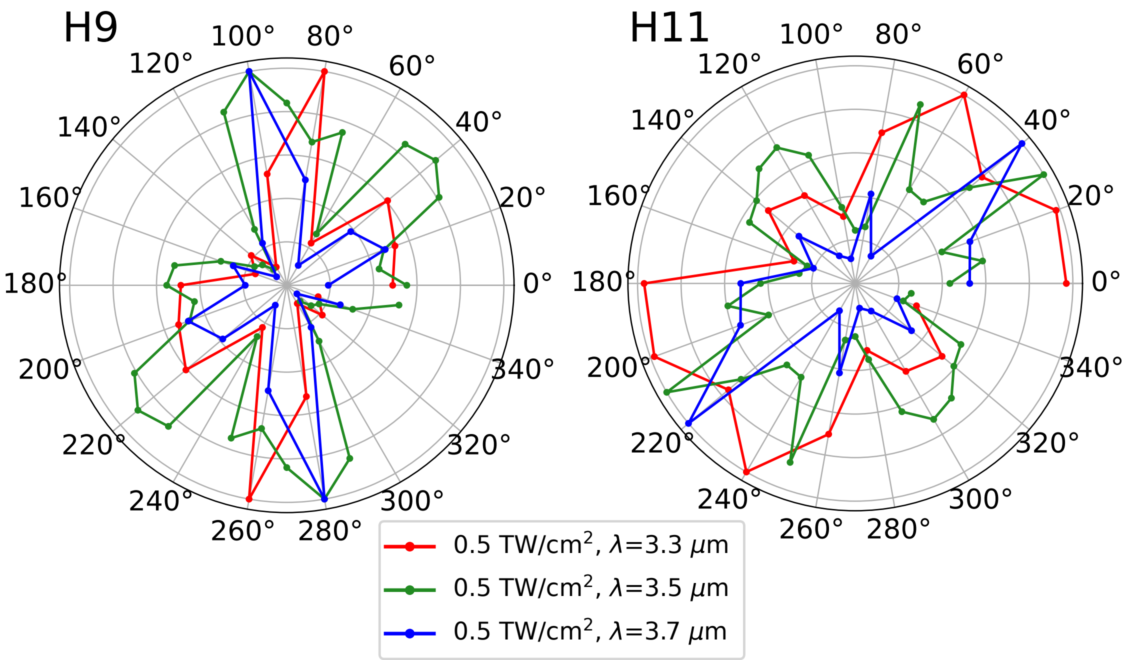

Supplementary Figure 14: Dependence of laser frequency (energy scales) on angle-resolved HHG in ReS_2. Simulated angle-resolved, energy-integrated spectrum for harmonics 9 (left) and 11 (right) for different central wavelengths: $\lambda = 3.3$ μm (red curve), 3.5 μm (green curve) and 3.7 μm (blue curve). The intensity is fixed at $I = 0.5$ TW/cm^2.
