# Supplementary material for: Orbital perspective on high-harmonic generation from solids
Source: Nat Commun. 2023 Dec 18;14:8421. doi: 10.1038/s41467-023-44041-0 (PMC10728088; doi:10.1038/s41467-023-44041-0)
Supplement: Supplementary file 3 — Description of Additional Supplementary Files [file 41467_2023_44041_MOESM3_ESM.pdf]

Description of Additional Supplementary Files

Supplementary Data 1 : Contains the centres of the Wannier functions that is outputted from the Wannier90 code
